# Antineutrophil Cytoplasmic Antibody (ANCA)-Associated Renal Vasculitis After COVID-19 Infection: A Case Report

**DOI:** 10.7759/cureus.26111

**Published:** 2022-06-20

**Authors:** Saurabh Kataria, Sylvette Rogers, Haleema Sadia, Tooba Ali, Hasham M Qureshi, Shehar Bano, Chinyere L Anigbo, Romil Singh

**Affiliations:** 1 Neurology, Ochsner Louisiana State University Health Sciences Center, Shreveport, USA; 2 Neurology and Neurocritical Care, University of Missouri Health Care, Columbia, USA; 3 Neurology, West Virginia University, Morgantown, USA; 4 Family Medicine, Caribbean Medical University, Atlanta, USA; 5 Internal Medicine, Khyber Medical College Peshawar, Peshawar, PAK; 6 Internal Medicine, Rawalpindi Medical University, Rawalpindi, PAK; 7 Internal Medicine, Isra University Hospital, Hyderabad, PAK; 8 Internal Medicine, University of Health Sciences, Lahore, PAK; 9 Internal Medicine, University of Nigeria, Enugu, NGA; 10 Critical Care, Allegheny Health Network, Pittsburgh, USA

**Keywords:** rheumatoid arthritis, antineutrophil cytoplasmic antibody (anca), anca associated vasculitis, covid-19, coronavirus disease

## Abstract

Antineutrophil cytoplasmic antibody (ANCA)-associated vasculitis is a class of autoimmune diseases that can cause kidney failure because of mononuclear cell infiltration and the destruction of small and medium-sized blood vessels. Coronavirus disease 2019 (COVID-19) may trigger or exacerbate autoimmune diseases. We present a case of ANCA-associated vasculitis in a patient with rheumatoid arthritis after a COVID-19 infection, who presented with intermittent hemoptysis and dyspnea and was diagnosed with COVID-19 pneumonia three weeks ago. Her clinical, radiological, and serological picture was concerned with pulmonary-renal syndrome. Her serum was positive for antinuclear antibody and ANCAs, and renal biopsy showed pauci-immune crescentic glomerulonephritis. She was diagnosed clinicopathologically with pauci-immune glomerulonephritis in the setting of rheumatoid arthritis (RA) after a COVID-19 infection. Her condition improved after she was treated with rituximab and pulse dose methylprednisolone.

## Introduction

Vasculitis induced by antineutrophil cytoplasmic antibody (ANCA) represents a group of small vessel vasculitides characterized by granulomatous and neutrophilic tissue inflammation, often associated with the production of antibodies against neutrophilic antigens [1]. ANCAs predominantly target myeloperoxidase (MPO) and leucocyte proteinase 3 (PR3). ANCA-associated vasculitis (AAV) may involve any body organ system, including the skin, renal, lungs, and brain [2]. Renal involvement in rheumatoid arthritis (RA) is a complex situation, and the events leading to the initiation of AAV are not well understood [3]. Various factors, including genetic factors, specific drugs, infectious agents, and environmental exposure, may trigger the autoimmunity and are involved in the pathogenesis of AAV [2]. Coronavirus disease 19 (COVID-19) among infectious agents has been documented to cause acute kidney injury. Although reports of renal involvement due to coronavirus disease have been described, an association between vasculitis and COVID-19 has been rarely documented [4]. We report a case of AVV in a female patient with RA after a COVID-19 infection.

## Case presentation

A 79-year-old woman with ischemic heart disease and rheumatoid arthritis (RA) presented with intermittent hemoptysis and dyspnea for the last three days. She was diagnosed with RA three years ago and was commenced on methotrexate and analgesia; however, she was not compliant with her RA medications. She also reported subjective weight loss. She was also admitted to the hospital three weeks ago for COVID-19 pneumonia, and she was discharged four days later after improvement in her condition.

**Figure 1:**
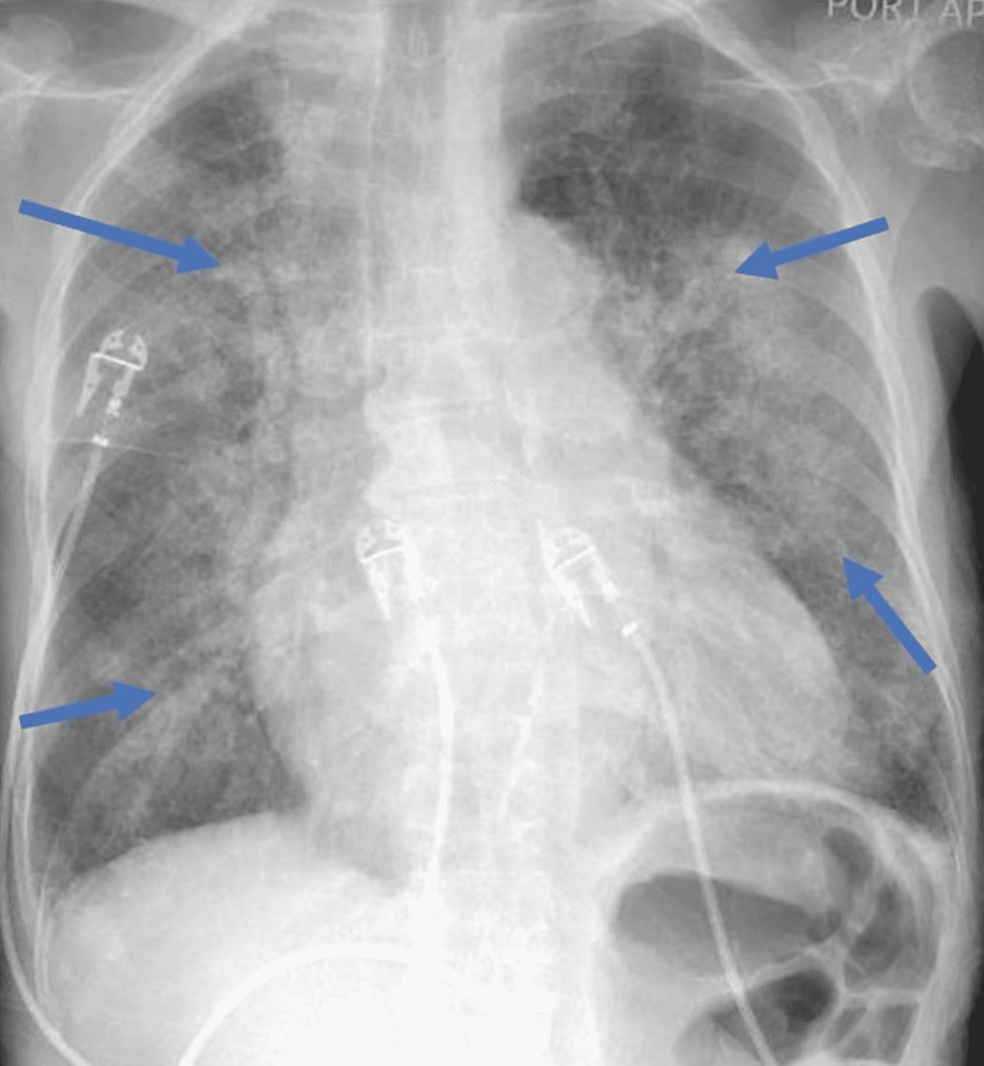
Chest X-ray showing bilateral patchy infiltrates and ground-glass opacities

On evaluation, she was afebrile, pale, and vitally stable except for hypoxemia (SpO2=90%). On physical examination, there were diffuse crackles and trace edema in the lower extremities. Laboratory studies were significant for low hemoglobin levels and increased levels of creatinine and brain natriuretic peptide (Table 1). Urinalysis showed hematuria, pyuria, and 1+ proteinuria. Her COVID-19 polymerase chain reaction (PCR) was positive for immunoglobulin G. A chest X-ray showed bilateral airspace disease (Figure 1). A ventilation-perfusion lung scan was negative for pulmonary embolism. She was diagnosed with volume overload and treated with furosemide 40mg twice daily with only partial improvement in respiratory symptoms but worsening renal dysfunction. Given her history, hemoptysis, hematuria, and worsening renal function, there was a concern for pulmonary-renal syndrome.

**Table 1:** Laboratory results on admission Hb: hemoglobin, WBC: white blood cells, BUN: blood urea nitrogen, ESR: erythrocyte sedimentation rate, BNP: brain natriuretic peptide, MCH: mean corpuscular hemoglobin, MCV: mean corpuscular volume, MCHC: mean corpuscular hemoglobin concentration.

Parameter	Lab value	Reference range
Hb	8.2	13-16 g/dl
MCV	82	77-95 fl
MCH	29	27-32 pg
MCHC	34	30-35 %
Platelet count	169000	150,000-350,000 cells/mm^3^
WBC	8900	4000-11,000 cells/mm^3^
Serum creatinine	2.4	0.7-1.2 mg/dl
BUN	41	13-21 mg/dl
ESR	31	0-22 mm/hr
BNP	2790	< 450 pg/ml

**Figure 2:**
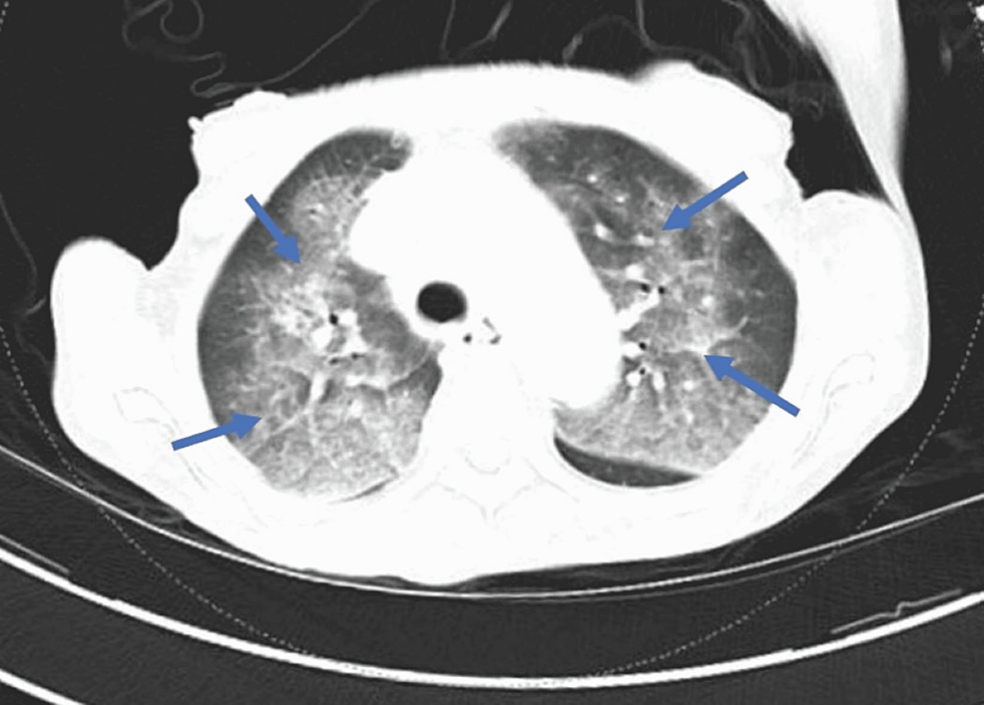
CT chest showing septal thickenings and patchy acinar opacities in the perihilar and central areas of both lungs

Additional pertinent studies included abdominal ultrasonography, which revealed normal kidney size and parenchymal echogenicity. Her serum was positive for ANCAs, and antinuclear antibodies (titer 1:160), and the staining pattern was homogenous and diffuse. Her serology titer for anti-MPO antibody (c-ANCA) was high (1:100), and for anti-PR3 antibody (p-ANCA) was 79 U/ml. She was negative for other autoantibodies except for the anti-cyclic citrullinated peptide (anti-CCP) antibody (Table 2). She underwent a renal biopsy which showed pauci-immune crescentic glomerulonephritis, diffuse global glomerulosclerosis, severe tubulointerstitial scarring, moderate to severe arteriosclerosis, and arteriolar hyalinosis. Her chest computed tomography showed acinar opacities and septal thickenings in the perihilar and central areas of both lungs (Figure 2). She was diagnosed clinicopathologically with pauci-immune glomerulonephritis in the setting of RA after COVID-19 infection. She was treated with rituximab of 1g and pulse dose methylprednisolone of 1g, followed by a tapering course of prednisone and a follow-up dose of rituximab. With follow-up, her serum creatine and proteinuria improved, and the lung findings regressed.

**Table 2:** Results of specific antibody testing dsDNA: double-stranded deoxyribonucleic acid, SM: smooth muscle, RNP: ribonucleotide protein, Anti-Scl-70: anti-sclerosis-70, CCP: cyclic citrullinated peptides.

Antibody screening	Status
Anti-dsDNA	Negative
Anti-SM	Negative
Anti-RNP	Negative
Anti-Scl-70	Negative
Anti-Ribosomal P	Negative
Anti-Centromere B	Negative
Anti-CCP	Positive

## Discussion

Renal involvement in rheumatoid arthritis (RA) is a complex situation. The most common causes of glomerular involvement in RA are secondary amyloidosis and medications, such as non-steroidal anti-inflammatory drugs and traditional disease-modifying antirheumatic drugs [5]. Overlap of RA and renal limited p-ANCA vasculitis is rare. Usually, p-ANCA vasculitis surfaces several years later, and infections can trigger the autoimmunity. However, limited literature is available to entertain the potential immune-mediated mechanisms resulting in various types of glomerular pathology, presentation, and treatment variabilities [6]. A prevalence of 21% of the overlap syndrome of RA and p-ANCA-vasculitis have been reported [7].

In COVID-19 and AAV, lung involvement is very common. Radiological picture of the patients with COVID-19 predominantly have ground-glass opacities distributed posteriorly and peripherally and consequent overlapping of consolidations, often accompanied by cavitation and pleural effusion [8]. In AVV, the patients have interstitial pneumonia or isolated ground glass appearance on imaging due to alveolar hemorrhage. Hence, clinical data and serology are recommended to distinguish COVID-19 from underlying autoimmune etiology [9]. We tabulated the reports of AAV following COVID-19 infection in table 3 [2,4,8,10].

**Table 3:** Summary of the published cases of AAV with COVID-19 M: male, F: female, GGOs: ground-glass opacities, AAV: antibody-associated vasculitis, ANCA: antineutrophil cytoplasmic antibody, NA: not available, DM: diabetes mellitus, S/Cr: serum creatinine, CP: cyclophosphamide.

Patient number	Izci Duran et al. [8]	Izci Duran et al. [8]	Moeinzadeh et al. [2]	Uppal et al. [10]	Jalalzadeh et al. [4]
Age/sex	26/M	36/F	25/M	64/M	46/F
Comorbidities	None	None	None	None	DM, Scleroderma
Peak S/Cr (mg/dl)	8.44	1.91	5.5	7.87	8.3
Serum albumin (g/dl)	2.58	2.22	NA	2.8	NA
Positive serology	p-ANCA	c-ANCA	c-ANCA	p-ANCA	p-ANCA
Chest X-ray	GGOs	Bilateral cavitary lesions	Alveolar hemorrhage	Bilateral patchy infiltrates	Pleural effusion, infiltrates
Kidney biopsy	Crescentic glomerulonephritis	Necrotizing crescentic glomerulonephritis	Crescentic glomerulonephritis	Crescentic glomerulonephritis	Crescentic glomerulonephritis
AAV management	Steroids, CP, plasmapheresis	Steroids, CP	Steroids, CP	Steroids, rituximab	Steroids, CP
Serum antibody level	p-ANCA: 80.6 U/ml	c-ANCA (1:32)	c-ANCA (1:50)	p-ANCA: 32.5 U/ml	p-ANCA (1:1280)

The pathophysiology of COVID-19 suggests that it may cause autoimmunity in genetically susceptible patients or exacerbate existing autoimmune diseases. Previous studies also verified that systemic bacterial or viral infections might lead to autoimmunity and cause pauci-immune granulomatous polyangiitis [11,12]. Somer et al. highlighted the reports of granulomatosis with polyangiitis (GPA) induced by cytomegalovirus and parvovirus-19 [11]. Another case report highlighted that GPA could be induced by superantigens of staphylococcus aureus by involving a specific autoimmune response, possibly by inducing a polyclonal response [12].

Pathophysiology of autoimmunity following COVID-19 infection may involve molecular mimicry, epitope spreading, bystander killing, viral persistence, and neutrophil extracellular trap formation. An elevated level of neutrophil extracellular traps has been observed in blood and kidney biopsy specimens of patients diagnosed with AAV [13]. These neutrophilic traps are pro-inflammatory proteins and may induce vasculitis directly by causing endothelial cell injury, complement system activation, and indirectly inducing the development of ANCAs [14]. Therefore, COVID-19 can trigger the emergence of autoimmunity or exacerbation of the autoimmune disease, leading to the development or flare of AAV.

Treatment with a chimeric anti-CD20 agent and steroids is an effective therapeutic approach in ANCA-associated vasculitis [1,5]. The prognosis of ANCA-associated renal vasculitis is poor unless addressed early in the disease course. The potential association between ANCA-associated vasculitis and RA is paramount, particularly in underlying infection and persistent microscopic hematuria and proteinuria [2,4,8]. It is challenging to know whether vasculitis is a complication or a flare of RA due to an underlying infection.

Our patient was diagnosed clinicopathologically with pauci-immune glomerulonephritis in the setting of RA after COVID-19 infection, who developed worsening of her renal and pulmonary functions, which was not caused by disease-modifying drugs. Her condition improved after commencing rituximab and pulse dose methylprednisolone, followed by a tapering course of prednisone and a follow-up dose of rituximab. Therefore, obtaining urine analysis and renal function tests in patients with RA is obligatory when serum creatinine level is high as a baseline and fails to improve despite appropriate interventions.

## Conclusions

Diagnosis of new-onset or flare of antibody-associated vasculitis in patients with RA following coronavirus disease is challenging. The infections, particularly COVID-19, may trigger the new onset or flare of vasculitis in individuals with RA, and a decline in renal function can help the differential diagnosis regardless of the pulmonary involvement. Immunological markers should be obtained in the presence of such clinical findings to assess or rule out vasculitis. Early recognition and appropriate management are mandatory to prevent or slow the progression of renal failure to end-stage renal disease.
